# Clinical and Economic Burden of Mental Disorders Among Children With Chronic Physical Conditions, United States, 2008–2013

**DOI:** 10.5888/pcd13.150535

**Published:** 2016-05-26

**Authors:** Manasi S. Suryavanshi, Yi Yang

**Affiliations:** Author Affiliation: Manasi S. Suryavanshi, Department of Pharmacy Administration, The University of Mississippi School of Pharmacy, University, Mississippi.

## Abstract

**Introduction:**

The prevalence of chronic physical and mental disorders is increasing among children and adolescents in the United States. In this study, we investigated the association between mental health disorders and chronic physical conditions among children, and we assessed whether having mental disorders is associated with increased health care costs for children with chronic physical conditions, using Medical Expenditure Panel Survey data from 2008 through 2013.

**Methods:**

Children aged 5 to 17 with at least 1 chronic physical condition were included in the study. Chronic physical conditions and mental disorders were identified using *International Classification of Diseases, 9th Revision, Clinical Modification* codes. We used logistic regression to assess the relationship between mental disorders and chronic physical conditions, and we used generalized linear models with gamma distribution and log link to estimate direct medical costs.

**Results:**

Of 42,130 children, 4,640 had at least 1 chronic physical condition. After controlling for sociodemographic and health care access characteristics, we found that children with at least 1 chronic physical condition were 62% more likely to have a mental health disorder than were children without chronic physical conditions (odds ratio = 1.62; 95% confidence interval [CI], 1.37–1.92). Having a mental disorder was a significant predictor of total health care cost (β = 0.64; 95% CI, 0.43–0.85; *P* < .001). The adjusted annual incremental cost due to mental disorders among children with chronic physical conditions was $2,631 (*P* < .001).

**Conclusion:**

Having chronic physical conditions in childhood is a significant predictor of mental health disorders and total health care expenditures.

**MEDSCAPE CME**

Medscape, LLC is pleased to provide online continuing medical education (CME) for this journal article, allowing clinicians the opportunity to earn CME credit.

This activity has been planned and implemented in accordance with the Essential Areas and Policies of the Accreditation Council for Continuing Medical Education through the joint providership of Medscape, LLC and *Preventing Chronic Disease*. Medscape, LLC is accredited by the ACCME to provide continuing medical education for physicians.

Medscape, LLC designates this Journal-based CME activity for a maximum of 1 ***AMA PRA Category 1 Credit(s)™***. Physicians should claim only the credit commensurate with the extent of their participation in the activity.

All other clinicians completing this activity will be issued a certificate of participation. To participate in this journal CME activity: (1) review the learning objectives and author disclosures; (2) study the education content; (3) take the post-test with a 75% minimum passing score and complete the evaluation at http://www.medscape.org/journal/pcd; (4) view/print certificate.

**Release date: May 31, 2016; Expiration date: May 31, 2017.**

**Learning Objectives**

Upon completion of this activity, participants will be able to:

1. Identify the association between mental health disorders and chronic physical conditions in US children, based on pooled 2008-2013 MEPS data
2. Assess the association between mental disorders and increased healthcare costs in US children with chronic physical conditions
3. Determine additional risk factors for mental health disorders in US children with chronic physical conditions

**EDITOR**

Camille Martin

Editor, *Preventing Chronic Disease*

Disclosure: Camille Martin has disclosed no relevant financial relationships.

**CME AUTHOR**

Laurie Barclay, MD

Freelance writer and reviewer, Medscape, LLC

Disclosure: Laurie Barclay, MD, has disclosed the following relevant financial relationships:

Owns stock, stock options, or bonds from: Pfizer

**AUTHORS**

Manasi S. Suryavanshi, MPH

Department of Pharmacy Administration, the University of Mississippi School of Pharmacy, University, Mississippi

Disclosure: Manasi S. Suryavanshi, MPH, has disclosed no relevant financial relationships.

Yi Yang, MD, PhD

Department of Pharmacy Administration and the Center for Pharmaceutical Marketing and Management, the University of Mississippi School of Pharmacy, University, Mississippi

Disclosure: Yi Yang, MD, PhD, has disclosed no relevant financial relationships.

## Introduction

During the past 50 years, the prevalence of chronic conditions among children has risen steadily in the United States. According to data from the 2011–2012 National Survey of Children’s Health, approximately 14% of US children had 1 chronic physical condition (1), which is an estimated 678% increase from 1960, when approximately 1.8% children younger than 17 years had a chronic physical condition (1,2). Advances in diagnostic procedures and management strategies may have contributed to the increase in the prevalence of childhood physical conditions. With appropriate medical care, many children with chronic physical illness are reaching adulthood. However, these children must adapt to their illness and cope with the difficulties and stresses inherent in living with chronic physical disorders. If left untreated, chronic stress can precipitate into various adjustment, emotional, and behavioral disorders among children, resulting in poor academic performance, which may adversely affect their employment success when they reach adulthood (3,4).

According to estimates from the Centers for Disease Control and Prevention, 13% to 20% of US children aged 18 or younger have some form of mental disorder (5), and an estimated 5 million children aged 5 to 17 receive treatment for mental health problems in a given year (6). Mental disorders are an independent predictor of poor clinical outcomes (7), and they are also among the most expensive conditions to treat among children aged 5 to 17; the average annual total cost for treatment from 2009 through 2011 was $10.9 billion (6,8). However, the clinical and economic burden of children having only a mental disorder may be substantially different from the burden of children who have coexisting physical and mental disorders. Children’s health is determined by the interaction between physical and mental influences; lack of proper management of either can result in deterioration of children’s overall health.

Chronic physical conditions in childhood may result in an emotional and economic burden on children and their families, because these children’s daily routines need to accommodate disease monitoring, visits to doctors’ offices, use of medications, and the use of medical devices (3). These conditions may limit children’s physical and social functions, putting them at a higher risk of behavioral disturbances and mental disorders such as anxiety, depression, and social withdrawal (9). The increased risk of having mental disorders may be linked with physical conditions other than just those involving brain functions (10). Some earlier studies attempting to understand the association between chronic physical conditions and children’s mental health focused on diseases such as asthma, diabetes, epilepsy, or sickle cell anemia (11–13). Children with chronic diseases share some common life experiences such as increased physician visits, hospitalizations, emergency events, complex medication regimens, days lost from school, and decreased social interaction with peers (14). The commonalities of many chronic physical conditions may contribute to the development of mental health disorders in children. To our knowledge, evidence is limited on the association between a range of debilitating childhood chronic conditions and children’s mental health. The incremental economic burden of mental disorders among children with chronic physical conditions in the United States is also inadequately documented.

The objectives of this study were to assess the association between mental health disorders and chronic physical conditions in children and to determine whether having mental health disorders is associated with increased health care costs for children with chronic physical conditions using a nationally representative sample of children aged 5 to 17 in the United States.

## Methods

### Study design and data

This study was a retrospective cross-sectional analysis of annual consolidated and event-level pooled data from the Medical Expenditure Panel Survey (MEPS) from 2008 through 2013. MEPS is a nationally representative survey of the US civilian noninstitutionalized population sponsored by the Agency for Healthcare Research and Quality (AHRQ). It provides information on individual and family health services use, health care expenditures and payment, and health insurance coverage. MEPS uses a complex sampling scheme that oversamples certain subgroups such as African Americans, Hispanics, and people from low-income households. A total of 80,784 families, or 203,282 individuals, provided data during 2008 through 2013 (15). Appropriate weighting techniques were applied during data pooling to account for the MEPS complex survey design and to adjust for people who appeared more than once in the pooled data set.

We used the household component of MEPS, which collects information from a single person in the family who has the most knowledge on family members’ health and health care use over 2 years. The information on health-related events and expenditures collected from households are supplemented by information from medical providers and pharmacies used by the households. Each individual’s event-level files and annual consolidated file are linked by a unique individual identification number.

A review by the institutional review board for this study was waived because the MEPS data are deidentified and publicly available to researchers.

### Subjects and measures

Children aged 5 to 17 who were noninstitutionalized members of the MEPS-responding households were included in the study. Data from MEPS 2008 through 2013 were pooled to obtain a sufficient sample for analysis. Children’s sociodemographic and health care expenditure data were obtained from each study year’s annual consolidated file. Information on children’s physical and mental conditions was obtained by using each year’s event-level files, including data on hospital inpatient stays, emergency department visits, outpatient visits, office-based medical provider visits, prescription medicine files, dental visits, and home health files.

Children’s chronic physical conditions and mental disorders were identified by using the *International Classification of Diseases, 9th Revision, Clinical Modification* (ICD-9-CM) codes, the Clinical Classification Codes (CCC) developed by the AHRQ, or both. Details on the list of chronic physical conditions, mental disorders, and the ICD-9-CM and CCC codes used to identify these disorders are in the Appendix.

Because children with chronic conditions experience similar life situations and difficulties regardless of their condition, we defined children with chronic physical conditions by viewing them as a whole rather than identifying them by disease (16). Specifically, children with at least 1 of the following physical conditions during the study year were categorized as having chronic physical conditions: juvenile rheumatoid arthritis, asthma, epilepsy, diabetes mellitus, infantile cerebral palsy, spina bifida, congenital anomalies of the heart, sickle-cell disease, cystic fibrosis, blindness or low vision, color or night blindness, hearing loss, and common childhood cancers (17).

Children were classified as having a mental health problem if they had at least 1 of the following mental disorder diagnoses during the study year: affective psychoses, anxiety, depression, conduct disorders, oppositional-defiant disorders, attention-deficit/hyperactivity disorders, personality disorder, acute stress, adjustment disorders, delusions, and other psychoses. The inclusion of mental disorders was based on the prevalence of mental disorders in children (18).

Total health care cost for each child was obtained by summarizing all event-level costs including costs for prescription drugs, emergency department visits, inpatient hospitalizations, out-patient visits, office visits, home health care, dental treatments, and other medical expenditures. Expenditures for over-the-counter medications and indirect payments unrelated to specific medical events such as the Medicaid Disproportionate Share Hospital cost were excluded from the total cost calculation. All costs were reported in 2013 US dollars adjusted for medical price indices. Each child’s sociodemographic information (age, sex, race/ethnicity) and access to care variables (health insurance status, poverty level, place of residence) were included as covariates in analysis.

### Analysis

Data management and statistical analyses were performed using SAS version 9.4 (SAS Institute, Inc) and Stata version 12 (StataCorp, LP). Bivariate analyses were conducted using student’s *t* tests for continuous variables and χ^2^ tests for categorical variables to assess baseline characteristics among children with chronic physical conditions. Multivariate logistic regression was used to assess the relationship between having chronic physical conditions and mental disorders in children. Based on the results of modified Park test and link test in Stata, generalized linear models with a gamma distribution and a log link function were used to quantify the incremental costs associated with mental disorders among children with chronic physical conditions (19,20). Individuals’ sociodemographic characteristics and access to care variables were accounted for in all adjusted analyses.

MEPS sampling weights were applied in all analyses accounting for MEPS complex sample design and nonresponse. The results were also weighted to represent the noninstitutionalized children’s population in the United States.

## Results

Out of 42,130 (weighted estimate: 53,670,034) children identified in the MEPS 2008–2013 pooled data set, 4,640 (weighted estimate: 6,180,191) had at least 1 chronic physical condition. Among those, 609 (weighted estimate: 863,064) had at least 1 mental disorder on the basis of ICD-9-CM and CCC codes. The sociodemographic and health care access characteristics for children with chronic physical conditions are in Table 1.

**Table 1:** Baseline Characteristics of Children With Chronic Physical Conditions, Medical Expenditure Panel Survey, 2008–2013

Characteristic^a^	Chronic Physical Conditions	Mental Disorder	No Mental Disorder	*P* Value
**Total**	4,640 (100)	609 (13.90)	4,031 (86.10)	.06
**Mean age, y (SE)**	10.98 (0.08)	11.73 (0.22)	10.86 (0.08)	<.001
**Sex**				
Male	2,714 (57.47)	407 (62.48)	2,307 (56.65)	.11
Female	1,926 (42.53)	202 (37.52)	1,724 (43.35)	
**Race/ethnicity**				
Hispanic	1,361 (19.03)	136 (13.17)	1,225 (19.98)	.004
Non-Hispanic white	1,526 (54.89)	253 (63.48)	1,273 (53.49)	
Non-Hispanic black	1,361 (18.44)	174 (16.20)	1,187 (18.80)	
Asian, non-Hispanic	183 (3.03)	14 (1.88)	169 (3.21)	
Other^b^	209 (4.61)	32 (5.27)	177 (4.51)	
**Health insurance status**				
Private	2,017 (59.02)	238 (58.24)	1,779 (59.15)	.76
Public	2,501 (37.99)	358 (39.40)	2,143 (37.76)	
Uninsured	122 (2.99)	13 (2.36)	109 (3.09)	
**Poverty level^c^**				
Poor	1,595 (21.67)	204 (21.22)	1,391 (21.74)	.99
Near poor	356 (5.46)	53 (5.72)	303 (5.41)	
Low-income	821 (16.17)	116 (16.58)	705 (16.10)	
Middle-income	1,095 (29.25)	140 (29.10)	955 (29.27)	
High-income	773 (27.45)	96 (27.38)	677 (27.47)	
**US geographic region**				
Northeast	887 (20.71)	131 (24.31)	756 (20.13)	<.001
Midwest	977 (22.03)	176 (30.06)	801 (20.72)	
South	1,737 (37.76)	219 (33.38)	1,518 (38.47)	
West	1,039 (19.50)	83 (12.25)	956 (20.67)	

Abbreviation: SE, standard error

a Values are expressed as no. % unless otherwise indicated. Percentages and standard errors are based on weighted data to represent the civilian noninstitutionalized US population. Data source: Medical Expenditure Panel Survey, 2008–2013.

b “Other race/ethnicity” includes American Indians, Native Hawaiians, and people who reported multiple races.

c Poverty level is defined as poor (family income <100% of the federal poverty line [FPL]), near poor (family income from 100% to less than 125% of the FPL), low-income (family income from 125% to less than 200% of the FPL), middle-income (family income from 200% to less than 400% of the FPL), and high-income (family income ≥400% of the FPL).

The unadjusted analysis of the association between having a chronic physical condition and a mental health disorder indicated that children with at least 1 physical condition were 76% more likely than children without a physical health condition to have a mental disorder (odds ratio [OR] = 1.76; 95% confidence interval [CI], 1.50–2.06). After controlling for sociodemographic and health care access characteristics, we found that children with at least 1 physical condition were 62% more likely to have a mental health disorder than were children without a physical illness (OR = 1.62; 95% CI, 1.37–1.92).

The odds of having a mental disorder were 49% less for girls than for boys (OR = 0.49; 95% CI, 0.43–0.57). Hispanics (OR = 0.40; 95% CI, 0.34–0.48), non-Hispanic blacks (OR = 0.53; 95% CI, 0.44–0.63) and non-Hispanic Asians (OR = 0.23; 95% CI, 0.15–0.34) were also significantly less likely to have a diagnosed mental disorder than were non-Hispanic white children. Compared with children with private health insurance, children with public health insurance such as Medicaid or CHIP (Children’s Health Insurance Program) were significantly more likely (OR = 1.75; 95% CI, 1.44–2.14) and uninsured children were significantly less likely (OR = 0.51; 95% CI, 0.35–0.75) to have a mental health disorder. Children from high-income families were 24% more likely (OR = 1.24; 95% CI, 1.01–1.55) to have a mental health disorder than were children from poor families (Table 2).

**Table 2:** Multivariable Logistic Regression Determining the Odds of Children with a Physical Disorder Developing a Mental Health Disorder, Medical Expenditure Panel Survey, 2008–2013

Characteristic	Adjusted Odds Ratio (95% Confidence Interval)	*P* Value
**Physical health status**		
No physical disorder	1 [Reference]	
At least 1 chronic physical disorder	1.62 (1.37–1.92)	<.001
**Age, y**	1.08 (1.06–1.09)	<.001
**Sex**		
Male	1 [Reference]	
Female	0.49 (0.43–0.57)	<.001
**Race/ethnicity**		
Non-Hispanic white	1 [Reference]	
Hispanic	0.40 (0.34–0.48)	<.001
Non-Hispanic black	0.53 (0.44–0.63)	<.001
Asian, non-Hispanic	0.23 (0.15–0.34)	<.001
Other^a^	0.92 (0.71–1.19)	.55
**Health insurance status**		
Private	1 [Reference]	
Public	1.75 (1.44−2.14)	<.001
Uninsured	0.51 (0.35–0.75)	<.001
**Poverty level^b^**		
Poor	1 [Reference]	
Near-poor	0.96 (0.75–1.23)	.76
Low-income	0.96 (0.80–1.16)	.67
Middle-income	1.07 (0.89–1.29)	.46
High-income	1.24 (1.01–1.55)	.04
**US geographic region**		
South	1 [Reference]	
Northeast	0.98 (0.80–1.19)	.82
Midwest	1.14 (0.94–1.39)	.19
West	0.67 (0.54–0.82)	<.001

a “Other race/ethnicity” includes American Indians, Native Hawaiians, and people who reported multiple races/ethnicities.

b Poverty level is defined as poor (family income <100% of the federal poverty line [FPL]), near poor (family income from 100% to less than 125% of the FPL), low-income (family income from 125% to less than 200% of the FPL), middle-income (family income from 200% to less than 400% of the FPL), and high-income (family income ≥400% of the FPL).

Total health care costs were significantly higher for children with a mental disorder than for children without a mental disorder (Table 3). The annual incremental total health care cost of having a mental disorder among children with a physical comorbidity was $2,874.57 (*P* < .001). Costs for prescription drugs, emergency care, and office visits were approximately 2 times higher for children with a mental disorder than for children without a mental disorder. Results from the adjusted cost analyses using the generalized linear model indicated that mental health status was a significant predictor of total health care costs (β = 0.64; 95% CI, 0.43–0.85; *P* < .001) for children with physical conditions after controlling for sociodemographic characteristics and access to health care. After adjusting for sociodemographic characteristics and access to health care, we found that, among children with chronic physical conditions, having a mental health disorder resulted in significantly higher total health care costs ($2,630.67; *P* < .001), prescription drug costs ($1,055.41; *P* < .001), emergency care costs ($77.25; *P* = .004), and office-based visit costs ($894.16; *P* < .001) compared with not having a mental health disorder (Table 3).

**Table 3:** Unadjusted and Adjusted Health Care Cost for Children With a Physical Disorder and No Mental Disorder and for Children With Mental and Physical Comorbidities, Medical Expenditure Panel Survey, 2008–2013

Health Care Cost, $^a^	Unadjusted Cost			*P* Value	Adjusted Mean Cost Difference, $ (SE)^b^	*P* Value
	Physical Disorder and Mental Disorder (N = 609)	Physical Disorder But No Mental Disorder (N = 4,031)	Unadjusted Cost Difference			
	Mean Cost, $ (SE)					
Total health care cost	6,494.06 (541.74)	3,619.49 (326.16)	2,874.57 (629.88)	<.001	2,630.67 (428.21)	<.001
Prescription drug cost	2,257.71 (214.58)	1,133.49 (208.98)	1,124.22 (295.96)	.02	1,055.41 (187.36)	<.001
Emergency care cost	237.86 (45.85)	121.05 (9.36)	116.80 (45.92)	.01	77.25 (26.96)	.004
Inpatient cost	721.23 (289.43)	512.34 (108.31)	208.89 (311.48)	.50	143.02 (226.79)	.53
Outpatient cost	463.16 (141.53)	389.81 (106.77)	73.35 (171.93)	.67	48.91 (113.71)	.67
Office-based visit cost	1,944.94 (211.10)	790.17 (83.19)	1,154.77 (224.39)	<.001	894.16 (118.38)	<.001
Home health care cost	386.64 (182.40)	183.77 (35.60)	202.87 (190.90)	.28	344.91 (261.09)	.19
Dental visit cost	426.52 (59.98)	421.81 (51.36)	4.71 (79.66)	.95	34.59 (51.33)	.50
Other medical cost	55.97 (7.90)	67.00 (7.38)	11.03 (11.23)	.32	−12.84 (13.15)	.33

Abbreviation: SE, standard error.

a Expenditures adjusted for inflation with the Consumer Price Index to reflect 2013 dollars.

b Cost difference adjusted for age, sex, race/ethnicity, health insurance status, poverty level, and geographic region.

## Discussion

A large proportion of US children are living with at least 1 chronic physical condition. Using nationally representative MEPS data from a sample of children from 2008 through 2013, we examined the association between a range of chronic physical conditions and mental health disorders. We also quantified the incremental health care cost for children with physical conditions with and without concomitant mental disorders. The results of our study show that having a chronic physical condition during childhood is a significant predictor of the concurrent presence of mental health disorders and total health care expenditures, even after controlling for demographic and access to health care characteristics. Concurrent presence of mental health disorders also leads to an incremental total health care cost of approximately $2,631 per child annually.

Findings from our study suggest that children with a chronic physical illness, regardless of which illness, are more likely to have coexisting mental health disorders than are their healthy peers. This finding could be because children with chronic physical conditions face experiences associated with treatment and disease management such as complex treatment regimens and frequent visits to doctors’ offices or other health care facilities. In addition, these children may also experience lifestyle changes, limitations in daily activities, and fear of early death. Some may also face emotional hardships because of stigmatization, isolation by peers, and low self-esteem due to academic difficulties. All of these experiences can lead to anxiety, stress, and psychosocial distress, which can be especially prominent during adolescence when physical and hormonal changes start to occur (9,17,21).

Our study found that Hispanic, non-Hispanic black, and non-Hispanic Asian children were less likely to have a diagnosis of mental disorder than were non-Hispanic white children, which could be the result of poor access to care due to lack of health insurance or residing in a region with limited health care facilities. Research suggests that a high percentage of Hispanic children do not receive medical care, which could contribute to the under-diagnosis of mental disorders in this population (22).

Our results also show that among children with chronic physical conditions, those with public health insurance such as Medicaid or CHIP were significantly more likely to have a diagnosed mental health condition and uninsured children were less likely to have such a diagnosis, compared with children with private insurance. In the United States, children with public insurance are primarily on Medicaid or CHIP, which is designed for low-income families (23). Although research shows that children of low socioeconomic status are more likely to have emotional and psychological distress (24), our results support that children on Medicaid or CHIP have reasonable access to health care services, including mental health services (22,25,26). Among all groups, uninsured children have the poorest access to medical care services; therefore, they are most likely to have undiagnosed medical conditions, including mental health disorders (22,25,26).

Childhood mental disorders impose a significant economic burden on children, family, and society. Davis estimated that the total cost of childhood mental disorders are approximately $10.9 billion each year in the United States (6). Childhood mental disorders also impose additional economic costs on children with chronic physical conditions. After controlling for sociodemographic and access to care covariates, we found that the incremental cost of mental health disorders among children with at least 1 chronic physical condition is $2,631 per child per year.

Evidence on the incremental economic burden associated with mental disorders in children with a chronic physical condition demonstrates the scope and magnitude of these problems. If not properly addressed, mental health problems among children often persist into adulthood and can result in negative social outcomes, such as unemployment, substance abuse, and criminal behaviors, as well as increased burden on social support and disability programs (27). As the cases of chronic physical conditions and mental health disorders increase for children and adolescents in the United States, it is essential to increase the awareness of the importance of providing timely and effective management of both physical and mental disorders for children (28). Prevention, early diagnosis, and treatment of mental health disorders are essential to the overall well-being of children. Clinicians and public health professionals treating children and adolescents with chronic physical conditions should be aware of the coexisting psychological and behavioral problems among these patients. In addition to managing chronic physical conditions, the medical team should monitor the psychological well-being of their patients and screen for mental health problems at regular visits.

Several limitations should be considered when interpreting the results of this study. First, MEPS uses a nationally representative sampling technique, making estimates from this study generalizable to noninstitutionalized children across the United States; conversely, the results of this study cannot be generalized to children institutionalized at psychiatric health facilities, community treatment facilities, and group homes or psychiatric nursing facilities. Second, the ICD-9-CM diagnosis codes used to identify physical and mental disorders were truncated at 3 digits in MEPS. Analysis using truncated codes prevented us from controlling for disease severity, which may bias the study results. Information on disease severity, disease duration, and treatment modality would be helpful for more reliable estimates. Finally, because the study design was cross-sectional, we cannot establish temporality or causality between physical conditions and mental disorders. Causal inferences between childhood physical conditions and mental health disorders and health care costs should be made with caution. Nonetheless, this study provides useful insights into the relationship between chronic physical conditions and mental disorders and the effect of mental health disorders on the total health care cost for children with chronic physical conditions in the United States.

Having chronic physical conditions was associated with having mental health disorders in a nationally representative sample of children in the United States. For children with physical conditions, adjusted total health care costs, prescription drug costs, emergency care costs, and physician office costs were significantly higher for those with mental disorders than for those without mental disorders. Results from this study should be most helpful to pediatricians, allied health professionals, and public health practitioners who are in direct contact with children and their families. In addition to providing disease-specific therapies, health professionals should learn more about the mental health needs of these children.

## Appendix List of Physical and Mental Conditions Included in the Study and Their Diagnosis Codes

**Table:**

Condition/Disorder	ICD-9-CM Code	Clinical Classification Code
**Chronic physical condition**		
Juvenile rheumatoid arthritis	714	202
Asthma	493	128
Epilepsy	345	83
Diabetes mellitus	250	49,50
Infantile cerebral palsy	343	82
Spina bifida	741	216
Congenital anomalies	746, 754, 755	213
Sickle-cell disease	282	61
Cystic fibrosis	277	56
Blindness and low vision	369	89
Color and night blindness	368	89
Hearing loss	389	94
Acute lymphocytic leukemia	204	39
Acute myelogenous leukemia	205	39
Brain tumors	191	35
Neuroblastoma	194	41
Wilms tumor/nephroblastoma	189	33
Lymphoma	200, 201, 206	38, 37
Rhabdomyosarcoma	171	21
Retinoblastoma	190	41
Bone cancers	170	21
**Mental disorder**		
Affective psychoses	296	657
Anxiety	300	651
Depression	311	657
Conduct disorders	312	65
Oppositional-defiant disorders	313	652
ADHD	314	652
Personality disorders	301	658
Acute stress	308	651
Adjustment disorders	309	655
Delusions	297	659
Other psychoses disorders	298	659

Abbreviations: ADHD, attention-deficit hyperactivity disorder; ICD-9-CM, *International Classification of Diseases, 9th Revision, Clinical Modification*.

## Post-Test Information

To obtain credit, you should first read the journal article. After reading the article, you should be able to answer the following, related, multiple-choice questions. To complete the questions (with a minimum 75% passing score) and earn continuing medical education (CME) credit, please go to http://www.medscape.org/journal/pcd. Credit cannot be obtained for tests completed on paper, although you may use the worksheet below to keep a record of your answers. You must be a registered user on Medscape.org. If you are not registered on Medscape.org, please click on the "Register" link on the right hand side of the website to register. Only one answer is correct for each question. Once you successfully answer all post-test questions you will be able to view and/or print your certificate. For questions regarding the content of this activity, contact the accredited provider, CME@medscape.net. For technical assistance, contact CME@webmd.net. American Medical Association’s Physician’s Recognition Award (AMA PRA) credits are accepted in the US as evidence of participation in CME activities. For further information on this award, please refer to http://www.ama-assn.org/ama/pub/about-ama/awards/ama-physicians-recognition-award.page. The AMA has determined that physicians not licensed in the US who participate in this CME activity are eligible for ***AMA PRA Category 1 Credits™***. Through agreements that the AMA has made with agencies in some countries, AMA PRA credit may be acceptable as evidence of participation in CME activities. If you are not licensed in the US, please complete the questions online, print the AMA PRA CME credit certificate and present it to your national medical association for review.

## Post-Test Questions

### Study Title: Clinical and Economic Burden of Mental Disorders Among Children With Chronic Physical Conditions, United States, 2008–2013

#### CME Questions

1. You are consulting for a US public health department regarding the association between mental health disorders and chronic physical conditions in children. According to the analysis of pooled 2008-2013 Medical Expenditure Panel Survey (MEPS) data by Suryavanshi and colleagues, which of the following statements would ***most*** likely appear in your report?
  - Children with physical conditions were 26% more likely to have mental health disorders than were children without chronic physical conditions
  - After controlling for sociodemographic and access factors, children with vs without chronic physical conditions were not significantly more likely to have mental health disorders
  - Some physical conditions were significantly more likely than others to be associated with mental health disorders
  - While managing chronic physical conditions, the medical team should monitor the patients' psychological well-being and screen for mental health problems at regular visits
2. According to the analysis of pooled 2008-2013 MEPS data by Suryavanshi and colleagues, which of the following statements about the association between mental disorders and healthcare costs in US children with chronic physical conditions is ***correct***?
  - Having mental disorders was not a significant predictor of total healthcare cost
  - Adjusted annual incremental cost due to mental disorders among children with chronic physical conditions was $1630
  - Mental health problems persisting into adulthood can significantly affect unemployment, substance abuse, and criminal behaviors
  - Emergency care costs were not significantly higher in children with vs children without mental disorders
3. According to the analysis of pooled 2008-2013 MEPS data by Suryavanshi and colleagues, which of the following variables is ***most*** likely an additional risk factor for US children with chronic physical conditions being diagnosed with mental health disorders?
  - Non-Hispanic white ethnicity
  - Hispanic ethnicity
  - Non-Hispanic black ethnicity
  - Lack of health insurance

**Table:** Evaluation

**1. The activity supported the learning objectives.**				
**Strongly Disagree**				**Strongly Agree**
1	2	3	4	5
**2. The material was organized clearly for learning to occur.**				
**Strongly Disagree**				**Strongly Agree**
1	2	3	4	5
**3. The content learned from this activity will impact my practice.**				
**Strongly Disagree**	** **	** **	** **	**Strongly Agree**
1	2	3	4	5
**4. The activity was presented objectively and free of commercial bias.**				
**Strongly Disagree**				**Strongly Agree**
1	2	3	4	5
