# Erratum, Vol. 13, May 26 Release

**DOI:** 10.5888/pcd13.150535e

**Published:** 2016-06-09

In the article “Clinical and Economic Burden of Mental Disorders Among Children With Chronic Physical Conditions, United States, 2008–2013,” the wrong footnotes appeared under Table 3. Corrections were made to our website on May 31, 2016, and appear online at http://www.cdc.gov/pcd/issues/2016/15_0535.htm. We regret any inconvenience this error may have caused.

